# Designing trustworthy, low-threshold care coordination: co-creating a hybrid CareCompass with rural informal caregivers

**DOI:** 10.3389/fdgth.2026.1823169

**Published:** 2026-08-03

**Authors:** Martin Ernst, Johannes Pflegerl, Doris Maurer, Andrea E. Schmidt, Christina Lampl, Eva Turk

**Affiliations:** 1 Center for Digital Health & Social Innovation, University of Applied Sciences St. Pölten, St. Pölten, Austria; 2 Ilse-Arlt Institute for Social Inclusion Research, University of Applied Sciences St. Pölten, St. Pölten, Austria; 3 Verein Kleinregion Waldviertler Kernland, Ottenschlag, Austria; 4 Competence Centre for Climate and Health, Austrian National Public Health Institute, Vienna, Austria

**Keywords:** care coordination, co-design, equity-by-design, hybrid digital–analogue systems, informal caregiving, participatory design, rural design, trust

## Abstract

**Introduction:**

As health systems across Europe face the dual pressures of environmental change and demographic aging, the need for a socio-ecological transformation that integrates health, equity, and sustainability has never been more pressing. Informal caregivers are central to long-term care systems, yet they often face time poverty, fragmented support, and unequal access to resources. These are conditions that can also constrain participation in health-promoting and climate-friendly everyday practices. This study reports a participatory co-design process that developed and iteratively refined *CareCompass* (PflegeKompass), a hybrid (digital–analogue) network-mapping and task-coordination concept for rural caregiving contexts in Austria.

**Methods:**

Using a Design Thinking framework, we conducted three sequential in-person workshops in the region Waldviertler Kernland with *n* = 13 stakeholders (informal caregivers, community “third-space” actors, health professionals, and regional management). Workshop artefacts (posters, prioritisation outputs, and annotated prototype flows) and field notes were synthesised through an artefact-centred design synthesis informed by inductive coding and participant prioritisation outputs.

**Results:**

Findings show that caregivers' primary challenge is coordination under uncertainty requiring timely access to information and resources, contingency management when support fails, financial strain, and cumulative planning effort. These insights translated into concrete equity-by-design needs: offline-first and low-threshold interaction using familiar calendar/list elements; role-based visibility and selective sharing to support privacy and prevent relational conflicts; and an onboarding model anchored in a locally trusted facilitator rather than a purely digital adoption. Prototype testing further revealed “trust and dignity” as design material, motivating a shift from a graphical network map to a list-based representation to avoid social comparison and shame.

**Conclusion:**

We contribute a transferable account of rural co-design for caregiving technologies, demonstrating how hybrid infrastructures, trust-sensitive representations, and explicit implementation conditions can be embedded early to support equitable, climate-resilient care coordination.

## 1 Introduction

The climate crisis is no longer a distant threat but a manifest health crisis that demands urgent, transformative approaches (1, 2). As health systems across Europe face the dual pressures of climate change and demographic aging, the need for a socio-ecological transformation that integrates health, equity, and sustainability has never been more pressing (3–5). To foster sustainable social change, these approaches must rely on participatory methods that engage vulnerable populations directly (6). Central to this societal shift is the role of informal caregivers (ICs), often the “next of kin”, who remain the backbone of the long-term care (LTC) system yet are frequently overlooked in the transition toward a climate-neutral society.

### 1.1 The care gap and the vulnerability of next of Kin

As populations age worldwide, the demand for LTC, which provides essential personal care and daily assistance services, is growing (7). Across OECD countries, this gap is largely filled by informal caregivers, with the OECD estimating that informal caregivers provide around 80% of long-term care support. This reliance is particularly stark for those aging at home, where 60% of care recipients report receiving assistance exclusively from informal sources rather than professional services (8). The intense provision of informal care is directly linked to declining mental health and reduced labor market participation, often forcing caregivers to cut their working hours or opt for early retirement (9). In Austria specifically, around 950,000 individuals (two-thirds of whom are women) provide unpaid care for relatives or neighbours, contributing an economic value estimated at €3 billion (10, 11). However, this critical workforce is under threat. With an average age of 62, many caregivers are in older ages themselves, managing care relationships that often span years. As multimorbidity increases the societal demand for care, the pool of available ICs is shrinking due to declining fertility rates and rising female labor market participation (12).

This widening “care gap” exacerbates the documented psychological and physical strain on caregivers, often leading to social isolation and chronic health issues (13, 14). Family caregivers, particularly in rural and underserved areas, face fragmented support networks and disproportionate mental load. Crucially, this burden acts as a barrier to sustainability; time poverty and stress significantly reduce both the willingness and ability of caregivers to adopt climate-friendly behaviours, such as active mobility or sustainable nutrition (15–17).

### 1.2 The intersection of health, climate, and equity

The World Health Organization (WHO) emphasizes that aging populations face disproportionate risks from environmental stressors, necessitating integrated health and social care policies (18). While the co-benefits of sustainable nutrition and physical activity for reducing greenhouse gas emissions are well documented, food systems alone account for roughly 30% of global emissions and active travel significantly reduces chronic disease risk (19), vulnerable groups are often excluded from these benefits due to a lack of time and resources.

Recently, the concept of a “well-being economy” has gained ground, formalized in the WHO's *Geneva Charter for Well-being* (20). However, strategies to ensure “human and planetary well-being” while reducing social inequities within welfare states remain underexplored in academic literature. Specifically, there is a lack of socio-technical interventions designed *with* and *for* caregivers that facilitate climate-neutral decisions without adding to their burden and digitalization often does not consider vulnerable groups such as informal caregivers.

This paper addresses this gap by presenting findings from our project which promotes a “Triple Win” of climate-friendly practices, social participation, and health promotion. Using a participatory Design Thinking (21) process in the rural Waldviertler Kernland region of Austria, we co-developed a solution with caregivers and professional stakeholders. The objective was to address inequities in access and coordination while enabling climate-friendly choices.

The resulting socio-technical solution—the BetreuungsKompass (CareCompass)—is a hybrid network-mapping and task-coordination prototype. It is designed to make support networks visible, reduce caregiver burden, and overcome barriers related to connectivity and literacy. By focusing on sustainable nutrition, active mobility, and inclusive spatial planning, the project demonstrates how the “Health in All Policies” framework can be operationalized to support the next of kin, ensuring that the transition to a climate-resilient society is both equitable and health-centred.

Participatory design and rural HCI scholarship further suggest that rural communities should not be treated merely as geographically peripheral or infrastructurally deficient versions of urban contexts. Rural HCI research has argued for explicitly defining and contextualising rurality, including local social relations, mobility conditions, institutional arrangements, digital infrastructures, and the meanings attached to place (22). Related design work calls for designing from the rural rather than merely for rural users, foregrounding rural practices, aspirations, constraints, and infrastructures as generative design resources (23). Earlier participatory design research in rural contexts similarly emphasises that design activities need to be anchored in local customs, communication practices, and community relationships (24), while co-creation research in rural communities highlights sustained community engagement, local intermediaries, and sensitivity to local knowledge barriers and implementation conditions as central to meaningful participation (25).

Building on this literature, our study treats the rural setting not as a background variable but as part of the design problem itself. In the context of informal caregiving, rurality shapes how support is accessed, who is trusted as an intermediary, how mobility and connectivity constraints are experienced, and how care networks can realistically be coordinated. This study therefore addresses a practical and scholarly gap: there is limited evidence on low-threshold, co-designed socio-technical solutions that help caregivers coordinate support and enable climate-friendly everyday choices without increasing burden, while also taking seriously the rural infrastructures and relational conditions through which such solutions must be implemented. The paper therefore examines how a participatory co-design/Design Thinking process can translate caregivers' needs and local stakeholder knowledge into concrete requirements and a feasible hybrid prototype.

Our aim was to develop and characterize a hybrid solution that makes support networks visible, reduces coordination load, and remains inclusive under constraints such as limited connectivity, varying digital literacy, and trust requirements. We ask: (1) Which coordination problems and equity-relevant barriers do informal caregivers identify during the co-design process? (2) Which design requirements and principles emerge, and how are they implemented in the CareCompass (BetreuungsKompass) prototype? (3) Which implementation conditions (e.g., trust-building, local onboarding, offline/low-tech use) are critical for viability in a rural setting?

This paper contributes (i) an empirically grounded account of a rural co-design process with informal caregivers and professional stakeholders, (ii) the CareCompass as a hybrid network-mapping and task-coordination prototype, and (iii) transferable equity-by-design principles, particularly an offline-first, low-threshold design logic supported by trusted local actors.

## 2 Methods

### 2.1 Study design

This study employed a participatory co-design approach structured by a Design Thinking framework to develop an inclusive, low-threshold socio-technical solution for care coordination in a rural setting. Design Thinking was selected because it explicitly supports an iterative progression from empathic problem exploration to collaborative ideation and rapid prototyping, enabling solutions to be shaped by users' lived constraints rather than by assumed “average” technology use (21). In line with participatory design scholarship, we treated caregivers and local professionals not as informants but as co-creators, using workshop-based “making” activities to externalize tacit knowledge, negotiate priorities, and translate needs into concrete design features that can be tested and refined (26). This emphasis is particularly relevant for informal caregiving contexts, where prior work shows that many digital solutions fail to fit everyday routines, assume stable connectivity, and insufficiently address adoption barriers such as limited digital confidence and high cognitive load (27).

The study was embedded in a project, which frames innovation as a “triple win” across health, climate and equity/participation. In practical terms, this framing informed the study design by treating inclusion and accessibility as first-order constraints throughout the co-design process rather than as *post-hoc* usability improvements. Accordingly, the design logic prioritized “equity guardrails” that are especially salient for underserved rural caregivers, such as offline or hybrid use, simplified interaction flows, and locally trusted points of contact for onboarding and support, so that participation and adoption do not depend on constant internet access or high digital literacy (28, 29). Consistent with rural participatory design and Rural HCI scholarship, we treated the rural setting as an active design context rather than as a neutral location in which the workshops happened. Local infrastructures, trusted intermediaries, mobility constraints, connectivity variability, and existing community relationships were therefore considered part of the design material and not merely external implementation conditions (22–25).

Consistent with the Design Thinking cycle, the overall study design comprised sequential co-design workshops with iterative refinement between sessions, allowing emerging requirements and feasibility considerations to be progressively integrated into a hybrid prototype concept. Within this framing, climate relevance was treated as a design objective at project level, but not as a mandatory discussion topic to be explicitly prioritised by participants in each workshop activity. This decision was made to avoid imposing additional normative expectations on informal caregivers who were already participating under conditions of substantial burden and time pressure. Accordingly, the co-design process prioritised participant-defined coordination needs, while climate-related functions were integrated only where they could support everyday routines without increasing cognitive or practical load.

### 2.2 Setting

The study took place in the rural micro-region Waldviertler Kernland in the southern Waldviertel area of Lower Austria, Austria. The region is an inter-municipal association comprising 14 municipalities and covers roughly 550 km^2^, reflecting a dispersed settlement structure typical of rural areas (30). In such contexts, support and care-related services tend to be distributed across multiple local actors and locations, which can increase logistical and coordination demands for caregivers; infrastructural conditions relevant to daily mobility and access also vary across the broader Waldviertel region (30). With respect to digital infrastructure, rural regions may experience heterogeneous connectivity and differing levels of access to high-speed internet; at the same time, Province of Lower Austria reports ongoing broadband expansion initiatives in the Waldviertel area, illustrating both the relevance of and variability in digital access conditions that shaped our attention to low-threshold, hybrid use scenarios (31). This provided a realistic context for developing and iteratively refining a hybrid solution intended to remain usable under varying connectivity and literacy conditions.

### 2.3 Participants & recruitment

Participants were recruited via purposive, stakeholder-based sampling to ensure that the co-design process integrated both lived caregiving experience and the local implementation perspectives required for a feasible rural solution. In line with co-design scholarship, we sought a heterogeneous group of “knowing” across everyday practice, frontline support, and local coordination in order to translate tacit needs into concrete design requirements and prototype decisions (26). Recruitment was organized through regional partners and existing local networks associated with the project context, enabling access to informal caregivers as well as community and municipal actors who could realistically influence later adoption and maintenance. To reduce participation barriers and acknowledge participants' time, participants received a Waldviertel voucher for each workshop. The workshops were explicitly framed as contributing to the development of a socio-technical solution for informal caregivers. Community, professional, and regional stakeholders were therefore included not as substitute users, but as “Third Space” and implementation actors who could contribute knowledge about local infrastructures, support pathways, and feasibility conditions.

In total, *n* = 13 stakeholders participated across the co-design activities: four informal caregivers with diverse caregiving situations (including distance caregiving, co-resident spousal care, and care for in-laws with dementia and mobility limitations), six community-embedded stakeholders from the local “Third Space” ecosystem (e.g., regional development, business owners, direct marketing, and regional coordination roles), two healthcare professionals (a community nurse and a nurse/nutrition trainer), and one additional regional climate-adaptation management representative. Taking an inclusive and participatory approach, eligibility did not require high digital proficiency; instead, we explicitly welcomed variation in digital experience to stress-test low-threshold and hybrid (digital and analogue) assumptions at the co-design stage, consistent with evidence that caregiver-facing technologies often fail when they presume stable connectivity, time availability, or high digital literacy (27). For reporting, we use role-based participant codes to preserve confidentiality while providing a transparent breakdown of the stakeholder constellation (Table 1).

**Table 1:** Participant roles, stakeholder groups, and recruitment targets (role-based codes).

Participant code	Stakeholder group	Role/affiliation (as represented in the workshops)	Caregiving situation (if applicable)
IC1	Informal caregiver	Distance caregiver	Provides care at a distance
IC2	Informal caregiver	Co-resident caregiver	Cares for partner in shared household
IC3	Informal caregiver	Family caregiver (proximal)	Cares for in-laws nearby; mother-in-law with dementia
IC4	Informal caregiver	Spousal caregiver	Cares for wife post-stroke; limited ability to leave spouse alone
TS1	Community/Third Space stakeholder	Chair of regional LEADER development organization	–
TS2	Community/Third Space stakeholder	Local service provider (hairdresser)	–
TS3	Community/Third Space stakeholder	Regional health coordinator + municipality interface	–
TS4	Community/Third Space stakeholder	Retail business (specialist shop) + health-promotion (“Tut gut”) interface	–
TS5	Community/Third Space stakeholder	KLAR! manager + regional “Regionskisterl” initiative	–
TS6	Community/Third Space stakeholder	Operator of local food hut + direct marketer	–
HP1	Health professional	Community nurse	–
HP2	Health professional	Registered nurse + nutrition trainer	–
RA1	Regional management	KLAR! manager (regional climate-adaptation management)	–

All small-group activities and plenary discussions were facilitated, moderated, and documented by members of the research team. Potential differences between caregiver and stakeholder perspectives were anticipated through moderated group work, plenary reflection, and prioritisation exercises. Because informal caregivers were the primary target group of the solution, their needs would have been weighted particularly strongly in cases of conflict, while all stakeholder perspectives were considered with regard to feasibility, local implementation, and sustainability. In practice, however, perspectives were largely complementary rather than conflicting: stakeholder contributions typically extended caregivers' accounts by adding implementation knowledge, available local resources, or constraints relevant to later adoption.

### 2.4 Co-design process (workshops 1–3)

The co-design process followed an iterative Design Thinking logic across three sequential in-person workshops, moving from needs elicitation and problem framing to ideation/prioritization and finally prototype testing. Each workshop lasted approximately three to four hours and was held in physical rooms in the Waldviertler Kernland region, organised locally by DM. The number of participants varied slightly across sessions because of illness, but most participants were present for most workshops; across the full process, *n* = 13 stakeholders participated. Conducting the workshops in locally organised regional rooms was intended to reduce participation barriers, support hands-on work with physical artifacts, and situate the design process in the local community context rather than in an external university setting.

All three workshops were attended by ME, JP, and ET as researchers. ME introduced the project framing, presented workshop inputs, and later presented the prototype materials. JP moderated plenary discussions and reflection rounds. ME, JP, and ET facilitated small-group activities, supported participants during hands-on exercises, and documented discussions, decisions, and artifacts through field notes and photographs of workshop outputs. DM organised the local workshop setting and supported regional coordination. This facilitation structure ensured that each small group could be accompanied by a researcher and that emerging insights could be documented throughout the sessions.

**Figure 1:**
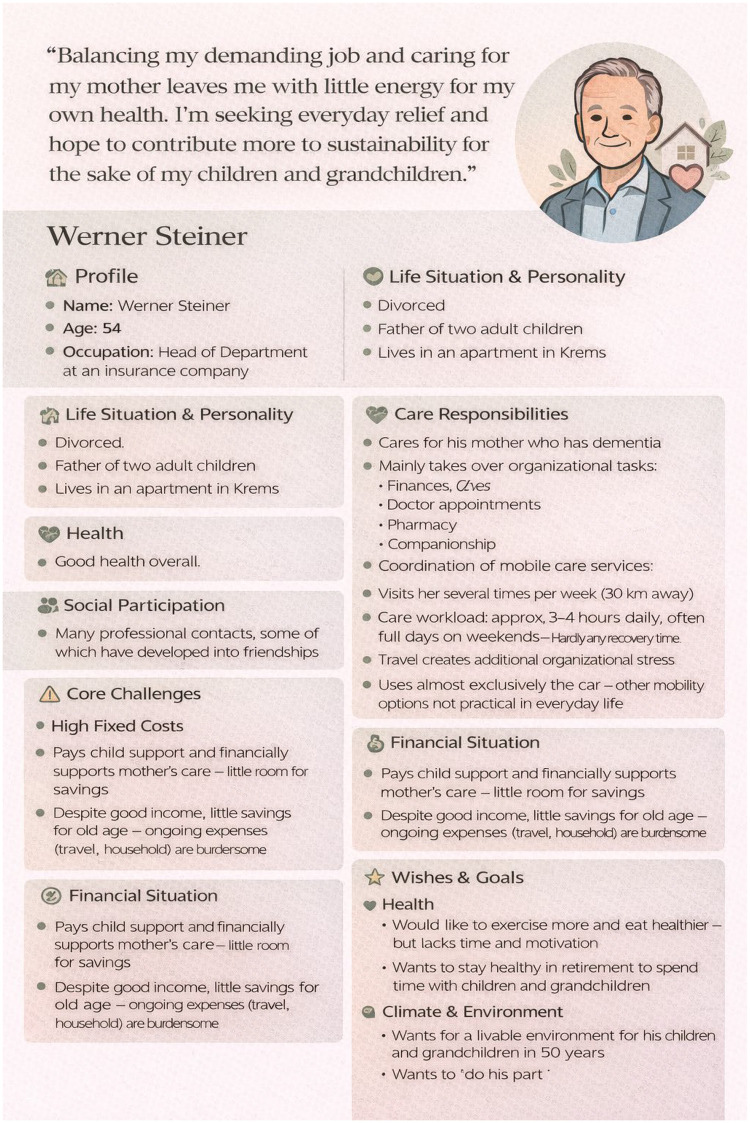
Persona “Werner” (distance caregiver) used in workshop 1 to support needs elicitation and day-journey mapping. Werner is a 54-year-old insurance professional who lives alone and provides ongoing support to his mother, who has dementia, while balancing full-time work and limited time for recovery. The persona highlights common constraints identified in the co-design process, including time poverty, high coordination load across errands and appointments, reliance on car-based mobility, and difficulty maintaining healthy routines and social participation. It also captures Werner's motivation to contribute to climate-friendly living, which is constrained by exhaustion and the practical demands of caregiving and everyday logistics.

**Figure 2:**
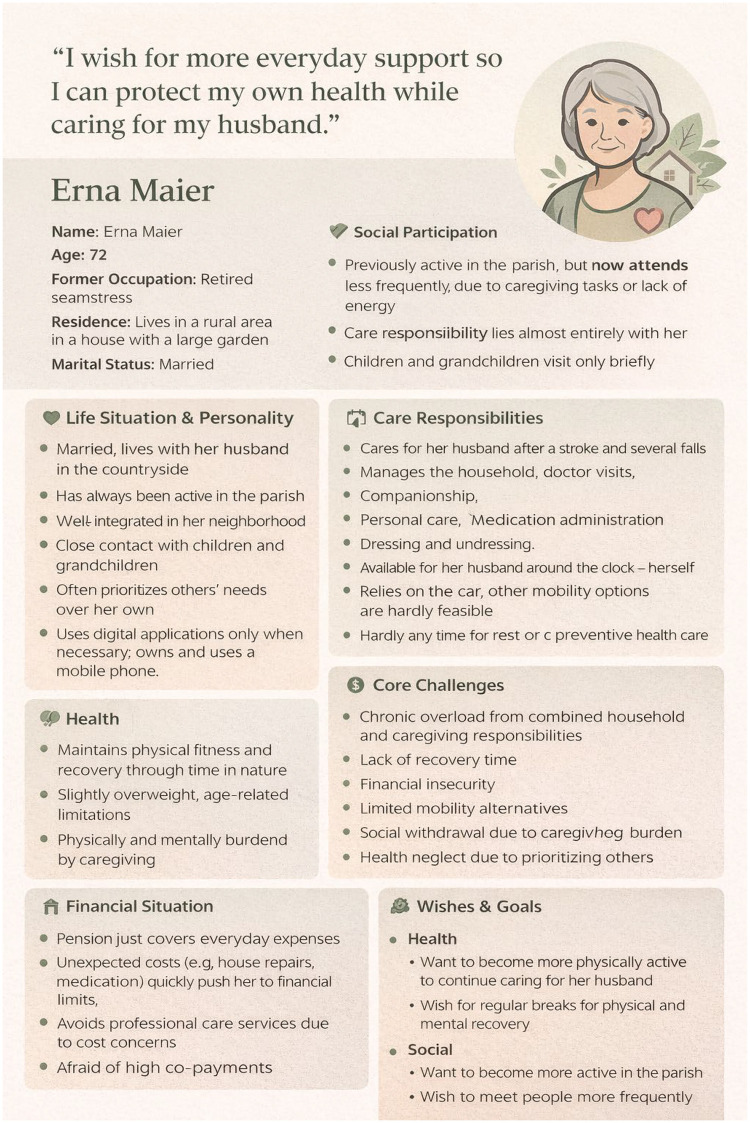
Persona “Erna” (co-resident spousal caregiver) used in workshop 1 to support needs elicitation and day-journey mapping*.* Erna is a 72-year-old retired seamstress living in a rural area with her husband, for whom she provides extensive day-to-day care following a serious fall and recurrent health limitations. The persona foregrounds common challenges in informal caregiving, including sustained physical and emotional workload, limited time and energy for self-care, restricted mobility and reliance on car-based errands, and heightened sensitivity to unexpected events. It also reflects Erna's desire for regular respite, continued social participation, and the ability to maintain climate-conscious practices in everyday life without neglecting caregiving responsibilities.

Workshop 1 (Empathize & Define) focused on surfacing constraints in caregivers' daily routines and pain points related to their social network. The session began with an introduction to the project aims, the participatory workshop logic, and the focus on a socio-technical solution for informal caregivers. Participants then engaged in perspective-taking exercises using two project-developed personas representing different caregiving scenarios: a distance caregiver and a co-resident spousal caregiver (depicted in Figures 1, 2). These personas had been developed in an earlier project phase based on focus groups with informal caregivers. We deliberately used individual personas rather than pre-defined care networks because the idea of a network-mapping and coordination tool emerged only later during the co-creation process. At the empathize-and-define stage, the aim was to foreground the everyday perspective, mental load, and vulnerability of a primary caregiver, who often acts as the main coordinating person in informal care arrangements.

The personas were designed to be concrete enough to support perspective-taking and discussion, but open enough to allow participants to project different local constellations, support gaps, and possible network resources into the exercise. For example, Erna's care responsibility was described as being carried almost entirely by her, while the wider support constellation was intentionally not fully specified. This openness was intended to stimulate discussion about who might realistically be involved, which forms of support might be missing, and how such support could be made visible or coordinated. Thus, the personas did not presuppose the later CareCompass network logic; rather, they helped surface the coordination needs from which this logic developed.

Working in small groups, participants constructed “typical day” maps for these personas to identify friction points regarding nutrition, mobility, time scarcity, access to support, and uncertainty in everyday care coordination. The resulting needs and barriers were clustered on workshop posters. Each participant then received three dot-voting points to prioritise the most urgent needs. The prioritised clusters served as the empirical input for the next workshop stage.

Workshop 2 (Ideate & Prioritize) translated these priorities into concrete solution concepts. The session began with a recap of the prioritised needs and barriers from Workshop 1. The research team then presented three initial solution directions derived from the first workshop outputs. Participants refined these concepts through a world-café format, rotating through stations to elaborate implementation possibilities, anticipate challenges, and suggest improvements. Concept and feature prioritisation combined participant voting, card-based prioritisation, and consensus discussion. Prioritisation was guided by recurring criteria that emerged across the workshop outputs: burden reduction for informal caregivers, feasibility under local resource constraints, low-threshold and hybrid usability, trust and controllable visibility, compatibility with existing local support structures, and alignment with the project aim of developing a digital or hybrid socio-technical solution. The final prioritisation indicated CareCompass/BetreuungsKompass as the strongest concept for prototyping because it most directly addressed coordination under time scarcity and uncertainty while also meeting the project requirement of developing a digital or hybrid solution. This decision was subsequently reflected on within the project consortium and confirmed for prototype development.

Workshop 3 (Prototype & Test) focused on validating and refining the selected concept. The session began with a short recap of the selected CareCompass concept and the rationale for the prototype. Participants then reviewed printed screen sequences of a clickable Figma prototype, covering both a network coordinator/admin flow and a network member/partner flow. The printed screen flows allowed participants to annotate the prototype directly, add sticky-note feedback, and discuss acceptance, perceived usefulness, trust, privacy, onboarding, and task-safety concerns. The session ended with a facilitated discussion of required changes, unresolved implementation issues, and conditions for local adoption. Feedback from this workshop was translated into the final prototype iteration.

### 2.5 Data sources & materials

Data collection focused on workshop-generated materials created during the co-design activities. Across the three workshops, the research team compiled field notes documenting plenary discussions and group work, and collected photographs of physical workshop outputs, including typical-day maps, posters, clustering materials, dot/sticker voting outputs, card-based prioritisation artifacts, and annotated prototype flows. In Workshop 1, the key artifacts were persona-based typical-day maps and clustered needs-and-barriers posters. In Workshop 2, the key artifacts were concept posters, world-café notes, card-sorting/prioritisation outputs, and voting materials used to compare and refine the three solution concepts. In Workshop 3, a clickable Figma prototype was tested in two role-specific flows (network coordinator/admin vs. network member/partner). Feedback was captured directly on printed screen flows via sticky-note annotations and subsequently consolidated alongside annotated prototype screenshots used to document change requests and refinements.

Selected anonymised artifacts are included in the manuscript and Supplementary Material to support transparency and traceability between participant contributions and design decisions. Identifying details were removed before inclusion. All materials were pseudonymized and processed in line with the institutional ethics framework of the University of Applied Sciences St. Pölten and the principles of data minimization and proportionality under the EU General Data Protection Regulation (GDPR). Access to the dataset was restricted to the research team (stored on a secure and encrypted university server). No continuous audio recordings were required for the study; instead, discussions and decisions were captured through written field notes and the visual/annotated workshop artifacts described above.

### 2.6 Data analysis

The analysis followed an artefact-centred design synthesis informed by inductive coding, using thematic-analysis principles as a sensitising reference for familiarisation, coding, and category development rather than as a stand-alone qualitative interview analysis (32). Because the study was embedded in an iterative co-design process, the analytical aim was not to conduct a stand-alone qualitative interview analysis, but to translate workshop-generated evidence into prioritised design requirements and prototype decisions. The primary data sources were field notes, photographed posters, dot-voting and card-prioritisation outputs, and annotated prototype screen flows. This approach is consistent with participatory design accounts that locate rigour not only in formal coding procedures, but also in transparent documentation of design decisions, reflexive discussion, and traceability between participant contributions, artifacts, and design outcomes (33). It also responds to calls for more explicit reporting of generative participatory design procedures in eHealth and socio-technical intervention development (34).

Analytically, ME first reviewed all workshop materials and field notes and conducted an inductive coding of recurring needs, barriers, feasibility concerns, and prototype-relevant requirements. The coding remained close to the workshop artifacts and prioritisation outputs rather than abstracting them into decontextualised themes. ET and JP then reviewed the coding structure, discussed the allocation of codes to broader design-relevant categories, and checked whether the proposed synthesis was traceable to concrete artifacts such as posters, voting outputs, card-sort materials, and annotated screen flows. Disagreements were resolved through discussion until consensus was reached. No formal interrater reliability was calculated because the analysis was interpretive and design-oriented rather than intended as a reliability-tested content analysis.

The synthesis produced two design output layers. First, Workshop 1 generated four prioritised needs-and-barriers clusters based on group mapping and dot voting: timely access to information and resources, financial strain, unpredictability and contingency management, and time burden/coordination load. Second, Workshops 2 and 3 generated prototype requirements and iteration clusters, including low-threshold language and framing, trusted-facilitator onboarding, role clarity and privacy-sensitive visibility, list-based representation of the support network, and task-safety boundaries for delegable activities. Throughout the process, the research team maintained an audit trail linking design requirements and prototype changes to specific workshop artifacts, prioritisation outputs, and documented discussions. Where prioritisation outputs produced equal or ambiguous support for competing concepts or features, the research team discussed these cases with consortium members to ensure that subsequent design decisions remained aligned with caregiver needs, local implementation constraints, and the overall project goal of developing a digital or hybrid socio-technical solution.

### 2.7 Ethics

This study followed University of Applied Sciences St. Pölten guidelines and EU GDPR principles. Formal ethics approval was not required for this voluntary, non-clinical co-creation research. Participants provided oral informed consent prior to workshops. Data were pseudonymized to ensure privacy, and reporting maintains participant anonymity using non-stigmatizing language.

## 3 Results

### 3.1 Needs and barriers: prioritised workshop clusters

**Figure 3:**
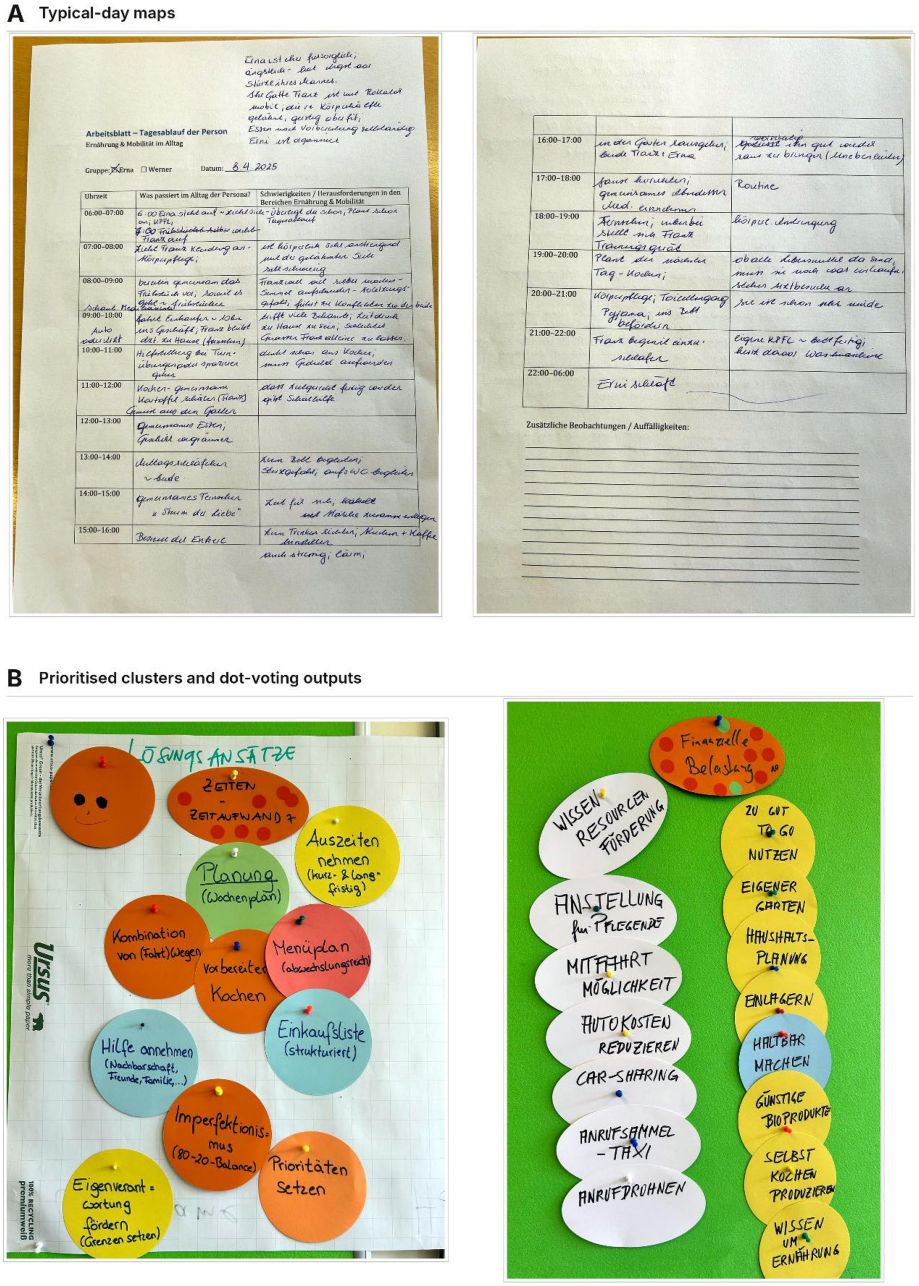
Anonymised workshop 1 artifacts illustrating the transition from persona-based day mapping to prioritised needs clusters. Panel A shows an example of a typical-day map constructed around one persona, documenting care-related tasks, errands, time pressure, mobility demands, and situations in which information or support was needed. Panel B shows the subsequent clustering and dot-voting output, from which the prioritised needs-and-barriers clusters were derived. Identifying details were removed before publication.

The needs-and-barriers findings were derived from participant-generated workshop artifacts rather than from interview transcripts. In Workshop 1, participants constructed typical-day maps around the personas and then clustered and prioritised the resulting friction points. The cluster labels and vote counts reported below therefore reflect documented workshop outputs, including posters, dot-voting results, and field notes. An anonymised example of a typical-day map and the associated prioritisation output is provided in Figure 3.

A first central cluster was timely access to information and resources, captured by the workshop cluster “Access to information & resources,” which received the highest vote count (17 points). Within this cluster, participants prioritised access to information at the right time (9 points) and the practical problem of finding relevant resources (7 points). This indicates that caregivers often know that support options exist but cannot retrieve them when they are needed or cannot translate generic information into actionable steps. Proposed solution directions documented on the workshop artifacts reinforced the same problem structure: an individualized, FAQ-like organization of information, a resource exchange for support such as shopping help or lending devices, and continuously available information for reassurance and guidance.

A second cluster concerned financial strain as an enabling or limiting condition for coping and for adopting healthier or more sustainable routines. “Financial burden” formed its own cluster with 10 points, indicating that costs were experienced not merely as a background factor but as a direct constraint on options such as mobility, food choices, or paid relief services. Associated artifact entries, including reducing car costs, carsharing, demand-responsive transport, affordable organic products, preserving food, and household planning, suggest that financial considerations were tightly coupled to both mobility feasibility and nutrition practices.

A third cluster was unpredictability and contingency management, summarized as “unexpected events,” also prioritised with 10 points. The corresponding artifact entries highlighted the unexpected failure of the caregiver or support person (7 points) and non-plannable events (3 points) as stressors that destabilize routines and trigger cascading coordination work. The associated coping ideas, including identifying who in the family is most reachable, activating neighbours for emergencies, and having clear pathways to 24-hour care or home nursing, point to the need for role clarity, rapid contactability, and predefined escalation routes within the support network.

A fourth cluster captured the time burden and coordination load of caregiving, operationalized in the cluster “time/effort,” which received 7 points and was repeatedly reflected in the personas' typical-day maps through continuous task switching, errands, and planning. Participants connected time scarcity to concrete barriers for nutrition and mobility, including limited opportunities for active mobility, the difficulty of quickly obtaining groceries, and special errands such as pharmacy or clothing trips, all of which add logistical overhead.

The associated solution ideas, including weekly planning, menu planning, combining routes, structured shopping lists, accepting help, and adopting an “80–20 balance” to reduce perfectionism, indicate that participants perceived planning tools and workload sharing as primary levers for reducing coordination load. These prioritised clusters directly informed the concept directions presented in Workshop 2 and explain why the later CareCompass prototype centred on shared planning, role clarity, task allocation, and low-threshold access to relevant information.

### 3.2 Concept and feature prioritisation

In Workshop 2, participants discussed and refined three competing solution concepts and then prioritised which concept should be taken forward into prototyping. This prioritisation was iterative and built directly on the Workshop 1 outputs. In Workshop 1, participants had identified needs and barriers through persona-based day mapping and then prioritised these through dot voting, with each participant receiving three voting points. In Workshop 2, these prioritised needs were translated into three concept directions and discussed through world-café stations, card-based refinement, voting, and a final consensus discussion.

The first concept, “Nahversorger+”, proposed a recurring low-threshold, community-based support point at local shops or similar venues (e.g., supermarket/market/pharmacy), combining brief advice, information materials, and social contact without requiring additional travel or digital access.

The second concept, a mobility and participation “Mitfahrbörse”, aimed to enable shared rides and joint participation in community activities via simple digital and analogue channels (e.g., web/app plus public postings), explicitly designed to work without registration and with accessible language and formats.

**Figure 4:**
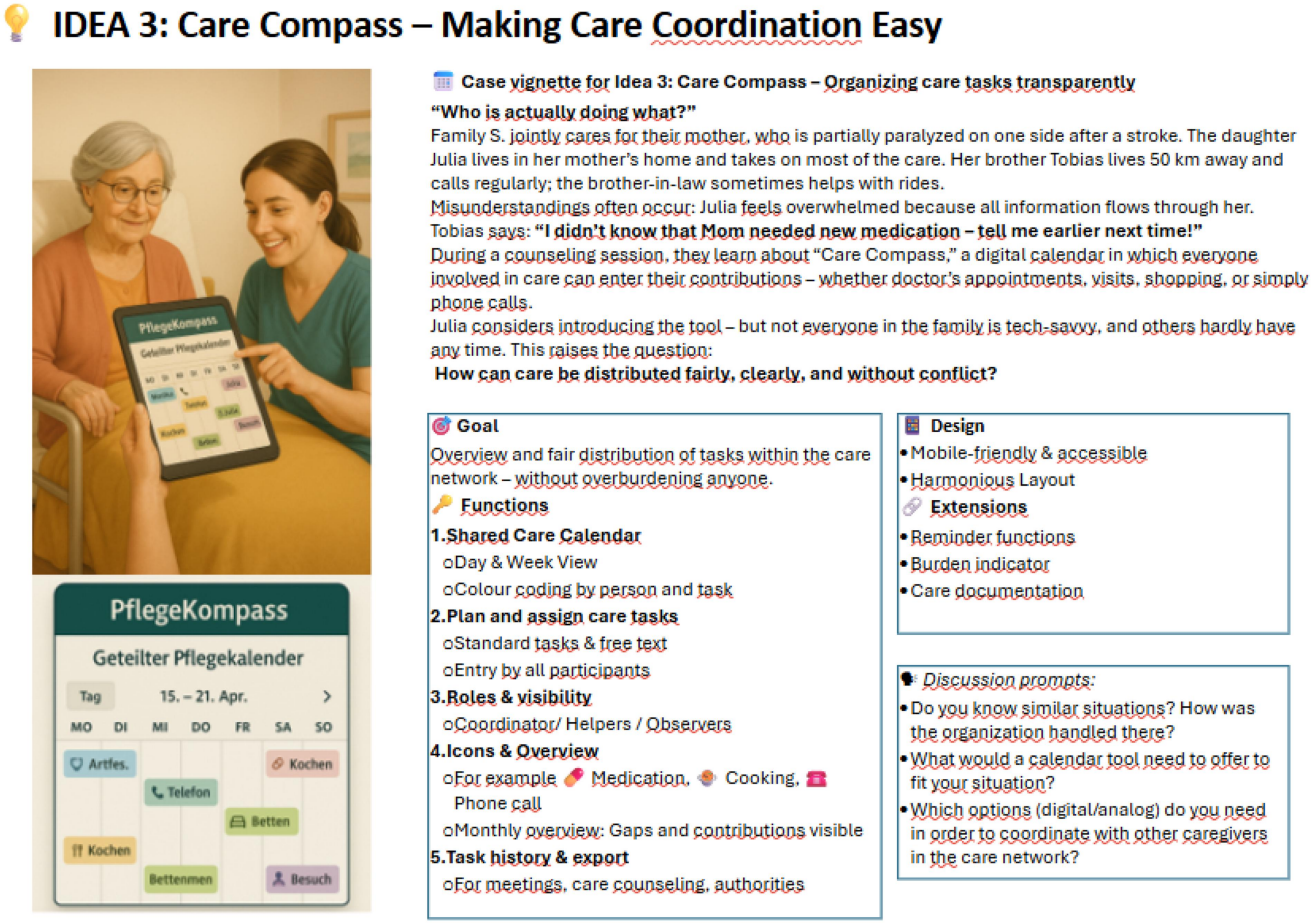
Carecompass (PflegeKompass) concept poster used in workshop 2 for idea refinement and prioritisation*.* The poster presents the CareCompass concept through a short scenario vignette that foregrounds common coordination breakdowns in family care and motivates the design question of how tasks can be distributed fairly and transparently. It summarizes the concept's core goal of reducing overload through shared coordination and outlines key functions, including a shared care calendar with day and week views, task planning and assignment, role-based visibility (coordinator, helpers, observers), and lightweight overview elements such as symbols and calendar-based at-a-glance structure. The poster also lists proposed design qualities and extensions, such as reminders, optional burden indicators, and documentation features, and ends with discussion prompts that guided group critique and selection during concept evaluation.

The third concept, CareCompass/BetreuungsKompass, focused on coordination within an existing helping network through a shared care calendar with role-based visibility and task allocation to reduce misunderstandings and perceived unfairness (“who does what?”) while keeping the interaction simple and familiar (Figure 4).

**Figure 5:**
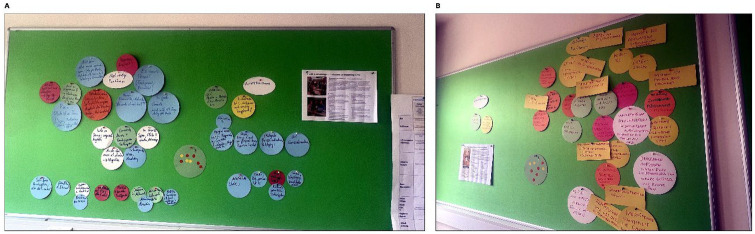
Workshop 2 concept-prioritisation artifacts documenting the transition from competing solution concepts to the selected CareCompass prototype direction. Panel A shows the workshop board used to collect notes, questions, and prioritisation points across the solution concepts (Nahversorger + concept in the example) discussed during the world-café and consensus process. Panel B shows the feature-level elaboration of the CareCompass/BetreuungsKompass concept, including participant-generated notes on necessary functions, a family coordinator role, free access, data security, recognisable tasks for multiple people, voluntary assignment options, role-based information visibility, reminder functions, and links to recurring care appointments or calendar structures. Together, the artifacts illustrate how the selected prototype direction emerged from participant-generated workshop outputs rather than from a researcher-defined concept alone. Identifying details were removed before publication.

Across the concept discussion, selection was guided by pragmatic criteria that repeatedly surfaced in the workshop outputs: feasibility under local resource constraints, burden reduction for the primary caregiver, low-threshold and hybrid usability, privacy and controllable visibility, compatibility with existing local support structures, and alignment with the project aim of developing a digital or hybrid socio-technical solution. Because the project explicitly aimed to develop such a digital or hybrid solution, CareCompass/BetreuungsKompass was prioritised over the other concepts: it most directly addressed the core problem structure identified in Workshop 1, namely coordination under time scarcity and unpredictability, while also offering the strongest pathway toward a concrete socio-technical prototype. The workshop board documented both the comparative discussion of solution directions and the feature-level elaboration of the selected CareCompass concept, including necessary functions, a family coordinator role, data security, role-based information visibility, recognisable tasks, assignment options, reminder functions, and calendar integration (Figure 5).

In particular, participants emphasized that any viable solution must not create additional workload for informal caregivers, volunteers, or partner institutions; should function with a stable and recognizable contact structure, such as a known community nurse or other trusted facilitator; and must allow caregivers to decide who sees what through role-based access and self-determined sharing. These criteria shaped the subsequent feature set. Core features were retained when they directly reduced coordination load, supported role clarity, could be represented through familiar low-threshold elements such as calendars or lists, and could be implemented without requiring constant connectivity or high digital literacy. Features were treated as optional or later-stage extensions when they were useful but less central to the coordination problem, more sensitive from a trust or liability perspective, or more dependent on local implementation conditions.

Participants highlighted concrete requirements that aligned naturally with the CareCompass logic, including a clearly defined family coordinator role, reminders for unassigned tasks, a printable version for the cared-for person, role-based visibility, and familiar calendar-like representations. Optional extensions, such as medication lists, meal planning, documentation features, or climate-related support modules, were retained as modular add-ons rather than as core functions. All three original idea posters can be found in the Supplementary Material S1.

### 3.3 CareCompass prototype

The CareCompass (BetreuungsKompass) prototype was conceptualized as a hybrid network-mapping and task-coordination tool intended to provide an overview of the helping circle and support fair, transparent distribution of care-related tasks.

Its core architecture combines three building blocks: (1) a network map to visualize the full support network (family, friends, neighbours, professionals) and to identify “silent resources,” (2) matching/task allocation to allow helpers to take on concrete tasks with clear feedback and commitment, and (3) a shared care calendar to structure day-to-day coordination and communication.

Functionally, the prototype includes a dashboard centred on the cared-for person with an overview of open tasks and currently active helpers, plus quick actions for entering tasks, requesting help, and messaging.

The matching logic supports task uptake (accept/decline), prioritization of open tasks, and reminders where gaps remain, aiming to enable engagement that is plannable and self-determined rather than based on pressure.

The care calendar provides day/week views, shared entries and assignments, and role-based visibility (e.g., coordination vs. helping vs. observing), with optional extensions (e.g., medication list, meal planning, bundling errands, mood/stress notes, “special observations” documentation) discussed as later add-ons rather than core requirements.

The network map allows role filtering, invitations of additional network members, and (optionally) visualization of activity/burden to support fair distribution; an export function was envisioned to support care counselling or planning conversations.

A defining design decision was the prototype's role logic, which was later implemented and tested via two distinct user flows: a network coordinator/admin perspective (setting up the network, entering/assigning tasks, managing visibility) and a network partner perspective (viewing relevant tasks, accepting contributions, providing feedback), consistent with participants' emphasis on explicit coordination responsibility while maintaining self-determined participation.

Hybrid, low-threshold requirements were reflected in features such as familiar calendar representations, controllable information visibility (“who sees what”), and the explicit expectation that outputs can be shared in print/offline form (e.g., a printed calendar for the cared-for person).

### 3.4 Prototype iteration insights—feedback and design changes

**Figure 6:**
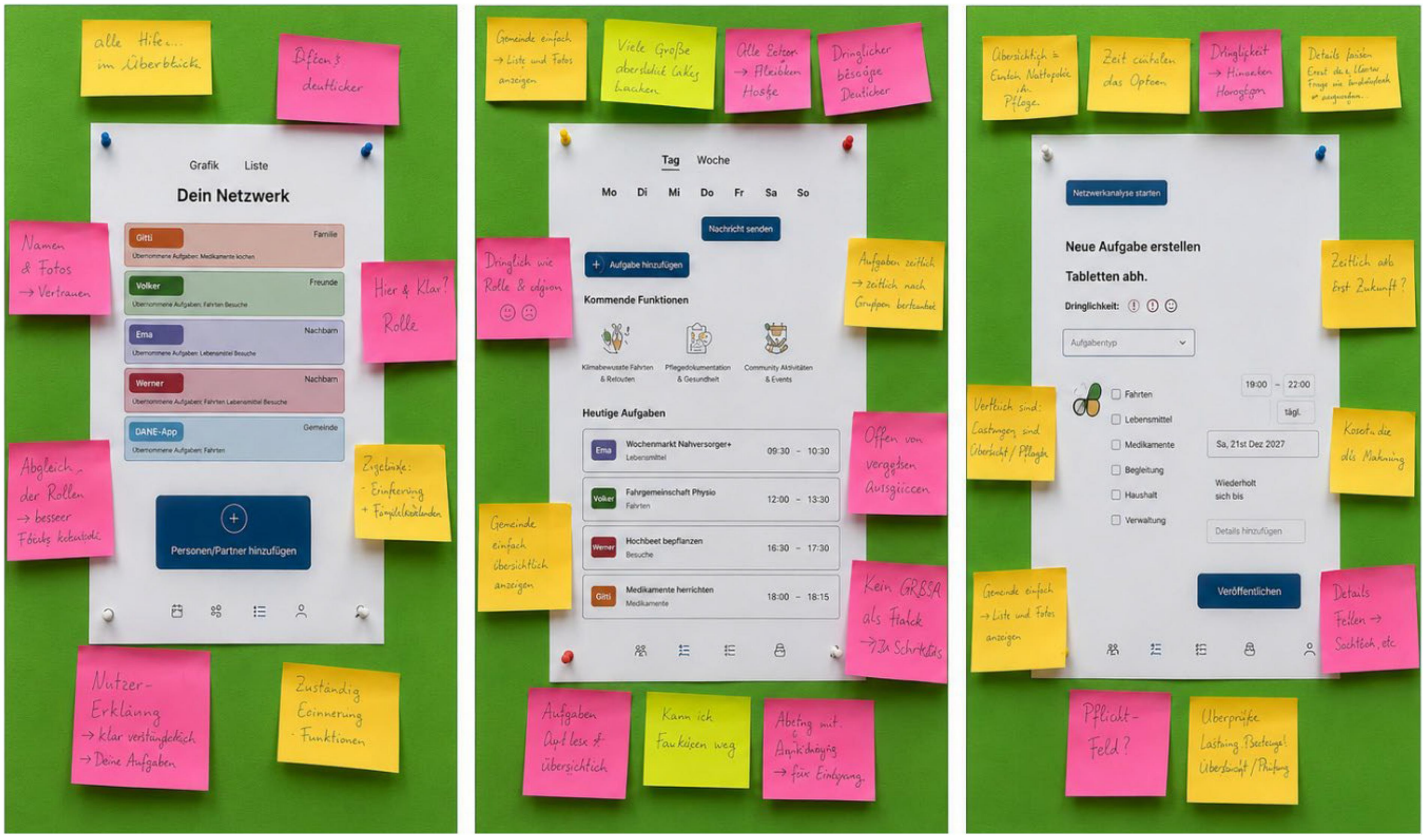
Anonymised workshop 3 prototype feedback artifacts. The figure shows examples of printed prototype screens annotated during the facilitated walkthrough. Participants used sticky notes and direct annotations to comment on clarity, privacy, role visibility, onboarding, and task-safety concerns. Identifying details were removed before publication.

Workshop 3 generated targeted feedback on clarity, trust and privacy, onboarding feasibility, and task safety, which was translated into concrete revisions from the first prototype to the refined version. The evidentiary basis for these changes consisted of annotated printed prototype screens, sticky-note comments, field notes from the facilitated walkthroughs, and the final plenary discussion. Figure 6 provides an anonymised example of annotated prototype materials showing how participant feedback was documented directly on printed screen flows and translated into prototype revisions.

A first cluster of changes addressed language and framing to reduce entry barriers for users with limited digital literacy. Participants noted that some labels felt overly technical or implicitly “app-like” and recommended simpler, more familiar wording as well as better readability. In response, the interface language was streamlined and reframed around a low-threshold “care calendar” and “helper group” concept, with simplified copy and improved legibility. This change was intended to reduce cognitive friction at first contact and to support inclusion beyond digitally confident users.

A second set of revisions focused on onboarding and the realism of support roles in rural regions. Participants discussed the idea that community nurses could serve as trusted onboarding helpers, while also highlighting that this role may not be available or recognizable everywhere. They therefore recommended an approach that can be extended to other locally anchored support roles such as link workers (35) or comparable coordination and counselling functions. The refined concept operationalized onboarding as a flexible “trusted facilitator” function rather than a single fixed professional category and strengthened low-threshold invitations through link-based joining and familiar communication channels. This improved implementation fit across heterogeneous service landscapes and reduced the risk of structural exclusion where a specific role does not exist.

A third line of iteration strengthened role clarity and privacy-sensitive visibility. Participants raised questions about who should act on behalf of the cared-for person, what helpers should be able to see, and how to avoid interpersonal friction within the network. The refined design therefore emphasized two distinct flows, one for the network coordinator or admin and one for network partners, and it introduced clearer role and permission configuration. It also followed a need-to-know logic, in which partners primarily see tasks relevant to them, while sensitive details can be withheld and certain appointments can be represented as blocked time without disclosure. These revisions were intended to increase trust, reduce unintended social tensions, and make participation feel self-determined rather than intrusive.

Related to these concerns, participants explicitly questioned how a visual network representation might be perceived. They noted that a graphical “network map” could feel exposing or even shaming for users with a smaller network and might invite social comparison rather than support (36). As a result, the refined approach favoured representing the network primarily as a list with categories or filters that foreground who can help with what, instead of highlighting network size through a diagram. This supported dignity and acceptability while keeping the representation functional for coordination and matching.

**Figure 7:**
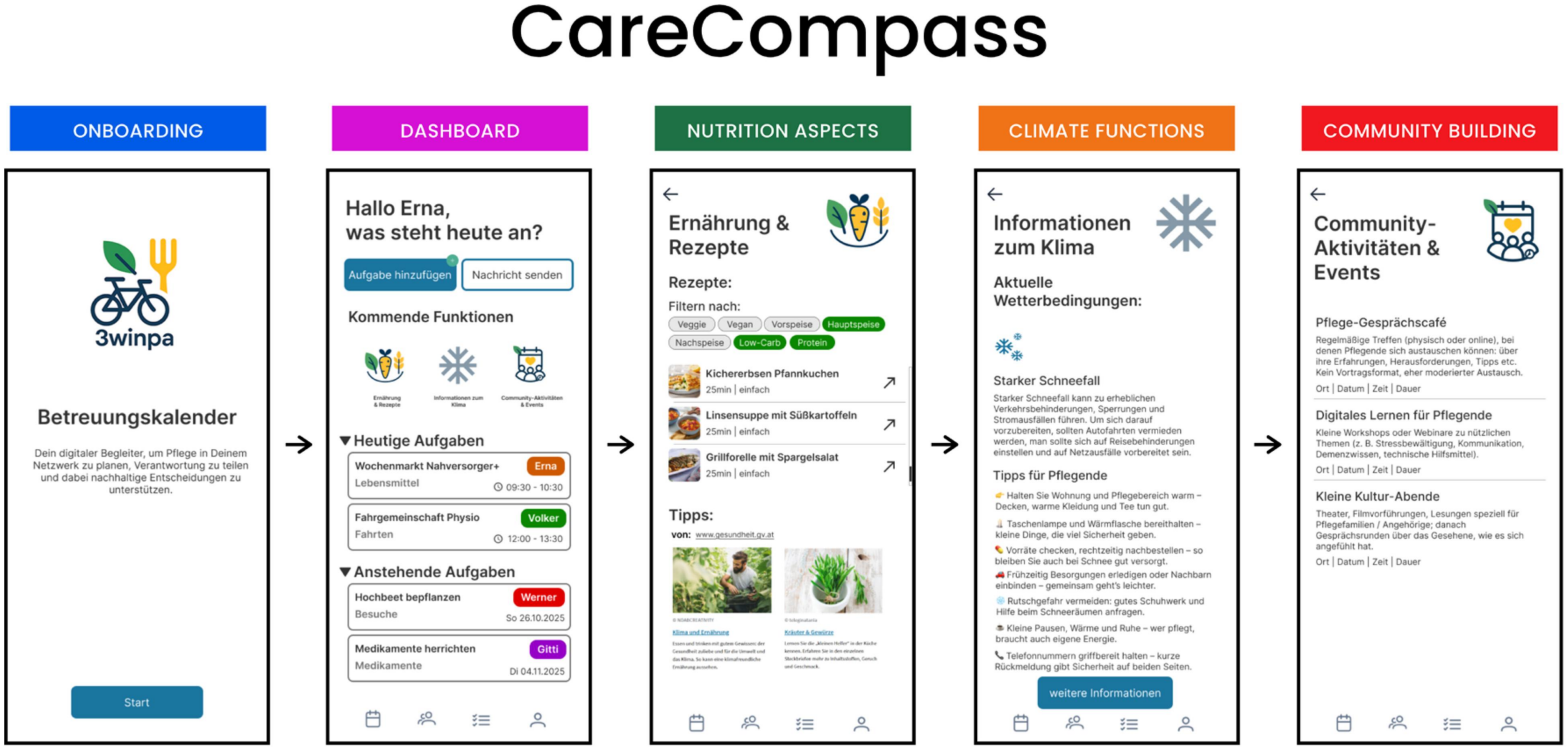
Refined CareCompass (PflegeKompass) prototype screens illustrating the core feature Set. The figure shows selected interface screens from the refined prototype, organised to reflect the main functional modules developed through the co-design process. It includes an onboarding entry screen and a caregiver-centred dashboard that provides an at-a-glance overview of current and upcoming coordination needs. The figure further depicts curated modules that extend the coordination tool with low-threshold support for everyday routines, including nutrition-oriented content (recipes and practical tips), climate-related information intended to support context-sensitive decision-making, and a community-building section that lists local activities and events relevant for caregivers. Together, these screens illustrate how the refined prototype combines a coordination backbone with modular supportive functions designed to remain usable under real-world constraints and varying levels of digital confidence (high resolution single screens are attached in Supplementary Material S2).

Finally, participants stressed that not all tasks are equally safe to delegate, with medication-related activities frequently cited as an example of potential risk and liability. They asked for clearer distinctions between care and support tasks including activities for daily living (ADL), instrumental activities of daily living (IADL) and for structured task descriptions that reduce ambiguity, including information about what is needed, when it is needed, and what constraints apply. In the refined version, task types and task wording were tightened toward safer, delegable actions and task-detail structures were made more explicit. This reduced the likelihood of inappropriate delegation and helped network partners contribute without requiring intimate clinical knowledge. In addition to the participant-prioritised coordination functions, the refined prototype also included modular climate-related support elements (e.g., context-sensitive information for extreme weather situations). These elements were not derived from a stand-alone climate theme in the workshop findings; rather, they were integrated as part of the project's Triple Win design framing (health, participation, climate) and adapted to the requirement that they must not increase caregivers' perceived burden. Central screens of the refined CareCompass prototype can be seen in Figure 7.

### 3.5 Equity-by-design outcomes

Across the co-design process and the prototype iteration, equity-by-design was operationalized through concrete guardrails rather than treated as an abstract claim. To reduce the cognitive load, the prototype employed low-threshold interaction logic, such as utilizing familiar metaphors (e.g, calendars, task lists) and simplified terminology. To accommodate regional heterogeneity, onboarding was designed around a flexible “trusted facilitator” model adaptable to various local actors beyond community nursing. Structural equity was further embedded by separating coordinator and partner workflows to distribute administrative burden, while role-based permissions ensured the selective visibility necessary for informal care networks. Additionally, a list-based network visualization was selected to mitigate social comparison and potential stigma regarding network size.

## 4 Discussion

### 4.1 Principal findings

The findings from the workshops indicate that climate-friendly routines were not articulated by participants as a primary or stand-alone concern. Their central challenge was the burden resulting from persistent coordination under time scarcity, high mental load, and uncertainty. Participants prioritised timely access to information and resources, contingency management when support fails, and the cumulative effort involved in planning and errands. This aligns with recent syntheses showing that caregiver-facing technologies often struggle when they do not fit fragmented routines and when they assume stable attention and high digital confidence (27, 37). It also echoes evidence that participatory methods remain inconsistently used and underreported in caregiver digital health interventions, which can weaken fit to lived constraints and implementation pathways (38). Importantly, this does not mean that climate considerations were absent from the intervention logic. In our co-design process, climate was not foregrounded as a stand-alone workshop topic in order to avoid adding normative pressure to an already highly burdened group. Instead, climate was treated as part of the project's Triple Win design framing and implemented in the prototype through explicit and implicit mechanisms, including context-sensitive support (e.g., extreme weather information) and coordination features that can facilitate lower-emission everyday decisions (e.g., combining trips or shared rides), while aiming to remain equitable and health-centred.

Second, concept selection suggested that the highest perceived leverage in this rural setting was reducing mental load through coordination clarity embedded within the caring communities-approach rather than offering a new standalone service. CareCompass was prioritised because it addressed role ambiguity and “who does what” tensions while remaining compatible with familiar representations such as calendars and lists. This reflects participatory design arguments that value is often produced through reshaping relations and responsibilities, not only through adding functionality, especially in care contexts where interdependence is the core condition (39).

Third, prototype iteration showed that trust and dignity are design materials that actively shaped interface decisions. Participants requested role-based visibility, selective sharing, and a list-based network representation because a graphical network display could make the size, density, or absence of a support network immediately visible. Based on the field notes, participants repeatedly raised the question of who would be able to see such a network and expressed concern that a sparse graphical representation could feel exposing or shame-inducing for caregivers with few available supporters. A list-based representation was therefore considered less socially evaluative and more pragmatic: rather than visually foregrounding “how many people” a caregiver has, it organises support around “who can help with what” through categories, filters, and task-relevant roles. In this way, the representation shifts attention from network size to actionable support, while role-based visibility and selective sharing further reduce the risk of unwanted comparison within the care network. Prior work has similarly argued that trust is socio-material and enacted through concrete choices in artifacts, interactions, and expectations rather than treated as an abstract attribute of “users” (40). In this study, trust requirements became visible precisely at the moment where coordination functionality risked becoming socially exposing. Fourth, equity constraints were operationalised as early guardrails that guided prototyping decisions. The refined design emphasised low-threshold language, hybrid joining via invitation links and familiar channels, and a facilitator-based onboarding model that could extend beyond community nursing to other locally anchored roles. This is consistent with broader participatory design accounts that stress infrastructuring and sustaining practices, which foreground long-term arrangements, support roles, and organisational conditions needed for initiatives to persist beyond project time (41). Rural design research also emphasises that connectivity variability and infrastructural unevenness are not edge cases but central design conditions that should be surfaced early (42).

### 4.2 Contribution to co-creation & participatory design

This case contributes to participatory design scholarship in three transferable ways. First, it provides an empirically grounded example of hybrid co-design in rural care that treats offline and low-threshold access as first-order requirements, addressing settings where “digital-first” assumptions can exclude those most vulnerable. In line with Rural HCI and rural participatory design scholarship, the rural setting was not treated as a deficit context or merely as a site of limited infrastructure, but as a design context shaped by local relationships, trusted intermediaries, mobility patterns, service availability, and uneven connectivity (22–25). The study therefore contributes to calls for designing from rural conditions rather than importing urban digital assumptions into rural communities.

Second, the study shows how trust can be built into socio-material arrangements through role logic, selective visibility, and representational choices, most concretely, shifting from a graphical network map to a list-based representation to reduce social comparison and support dignity in relational systems (39, 40). Finally, it advances an equity-by-design stance by linking prototyping decisions to explicit constraints (e.g., facilitation, inclusion, low literacy), consistent with participatory design's concern for the politics of participation and for locally anchorable, maintainable arrangements (41, 43). The contribution is therefore not only the CareCompass prototype itself, but also a documented design pathway showing how rural care coordination requirements can be translated into a trust-sensitive, low-threshold socio-technical concept.

### 4.3 Limitations

The findings are based on a small purposive sample in a single rural micro-region and may reflect region-specific service structures, social norms, and existing networks. Recruitment through established local partners may have favoured participants already engaged with community initiatives, which can limit transferability to more isolated or intensive caregivers. In addition, the initial empathize-and-define activities used personas centred on highly burdened primary caregivers rather than fully pre-defined care networks. This choice was appropriate for foregrounding the mental load and vulnerability of the main coordinating caregiver, but it may also have shaped the resulting prototype toward care situations in which one person carries substantial coordination responsibility. Future field testing should therefore include more distributed care networks, constellations with stronger professional involvement, and cases in which responsibility is shared more evenly across family members, neighbours, or community actors.

The study evaluated a prototype concept through workshop walkthroughs rather than measuring behavioural or health outcomes, and it did not include longitudinal adoption data. The prototype stage also means that some critical questions, such as governance agreements, legal liability boundaries, and operational financing, remain only partially addressed. Workshop feedback highlighted systemic barriers beyond interface design. Participants identified unresolved governance issues, including data ownership, service maintenance, and sustainable financing of regional support. Legal and trust-critical concerns regarding consent, decision authority, and liability for delegated tasks also persisted. These findings indicate that feasibility depends not only on technical refinement but also on the establishment of operational policies and regional agreements. A further limitation is that climate-related preferences and trade-offs were not systematically elicited as a dedicated topic in the workshops. This was an intentional design decision to avoid overburdening participants, but it means that the acceptability and perceived relevance of specific climate-related modules should be examined more explicitly in future field testing.

### 4.4 Future work

The next step should be a pilot or field test in which CareCompass is used in real caregiving networks over several weeks. Evaluation should combine coordination outcomes and burden proxies, including perceived coordination load, frequency of last-minute failures, task uptake rates, response times for support requests, and subjective confidence that help is available when needed. Such measures are consistent with the broader caregiver technology literature that emphasises usability under burden and fit to routines as determinants of sustained use (27, 37). Future iterations should also test onboarding models across regions that differ in service availability, comparing community nursing-led facilitation with other trusted local roles.

For transfer and scaling, research should focus on what must remain stable and what can be localised. The stable core likely includes role-based visibility, low-threshold task coordination, and hybrid joining. Localisable components include service directories, facilitator roles, and governance rules. Rural design work suggests that infrastructural variability should be treated as a design dimension rather than noise, so transfer studies should explicitly sample regions with differing connectivity and care service density (42).

Field deployment should explicitly test implementation conditions rather than assume them, especially credible local hosting, ongoing maintenance, and facilitation-based onboarding models (41). Pilot work should also co-define context-appropriate data governance (e.g., visibility rules, consent, and representation authority), as acceptable practices are often locally shaped rather than derivable from generic compliance statements (44). Finally, evaluation should examine potential unintended effects such as workload shift to primary caregivers and the need to operationalise task safety boundaries and escalation pathways in real-world use.

### 4.5 Conclusion

This study showed that supporting informal caregivers in climate-resilient transitions requires reducing coordination load under uncertainty rather than adding new demands. Through participatory co-design in a rural region, stakeholders prioritised a hybrid coordination concept and shaped a CareCompass prototype that embeds equity and trust constraints in concrete interface and role decisions. The resulting design makes coordination feasible under variable connectivity, uneven service availability, and sensitive trust dynamics. These insights matter because equitable climate-resilient care depends on socio-technical arrangements that respect caregivers' limited bandwidth while enabling healthier and more sustainable everyday choices.

## Data Availability

The raw data supporting the conclusions of this article will be made available by the authors, without undue reservation.
