# Supplementary material for: Designing trustworthy, low-threshold care coordination: co-creating a hybrid CareCompass with rural informal caregivers
Source: Front Digit Health. 2026 Aug 3;8:1823169. doi: 10.3389/fdgth.2026.1823169 (PMC13478116; doi:10.3389/fdgth.2026.1823169)
Supplement: Supplementary file 1 [file Table1.docx]

**Designing Trustworthy, Low-Threshold Care Coordination: Co-Creating a Hybrid CareCompass with Rural Informal Caregivers – Supplementary Material**

**S1** *Original personas Werner & Erna used in workshop 1*

**S2** *Original idea posters that were used in workshop 2 for the concept selction*

**S3** *High resolution Figma screens shown in Figure 4*
